# Rhizosphere Functional Plasticity and the Keystone Taxon *Sphingomonas* Facilitate Sweet Cherry Adaptation to Semi-Arid Stress

**DOI:** 10.3390/plants15111632

**Published:** 2026-05-26

**Authors:** Liyan Zhang, Jinyang Dong, Jun Zhao, Haiyan Jiang, Wenbing Zhang

**Affiliations:** 1 College of Forestry, Inner Mongolia Agricultural University, Hohhot 010019, China; zhangliyan@imau.edu.cn (L.Z.); igl@msn.com (J.D.);; 2 College of Horticulture and Plant Protection, Inner Mongolia Agricultural University, Hohhot 010010, China

**Keywords:** *Prunus avium*, sweet cherry, semi-arid environment, rhizosphere microbiome, *Sphingomonas*, stress-repair pathways, structural equation modeling, community assembly

## Abstract

Translocation of elite cultivars across distinct climatic regions often induces transplantation shock. Although the rhizosphere microbiome can facilitate host acclimation, the underlying functional mechanisms remain unclear. Here, we investigated microbiome-mediated adaptation in “Hongdeng” sweet cherry (*Prunus avium* L.) moved from a humid coastal region (Dalian, DL) to a semi-arid inland habitat (Hohhot, HS). We integrated plant physiological assays, metagenomic sequencing, and structural equation modeling (SEM) to compare the source population (DL), the introduced population (HS), and a locally acclimated reference cultivar (“Summit”, HSY). The introduced trees adjusted physiologically to the semi-arid environment by elevating proline levels and antioxidant enzyme activities. Although environmental stress reduced microbial alpha diversity, the core taxonomic framework persisted. Community assembly analysis indicated that the semi-arid climate intensified environmental filtering. Network analysis identified *Sphingomonas* as a keystone taxon; notably, it maintained a highly connected topological role despite a stable relative abundance. Furthermore, structural equation modeling showed that the environmental stress index positively correlated with the upregulation of microbial DNA repair pathways (*R* = 0.81, *p* < 0.001). Ultimately, the SEM demonstrated that environmental stress primarily shapes microbial functional profiles rather than driving species turnover, thereby contributing to host adaptation. The successful establishment of introduced sweet cherry in semi-arid regions is tied more closely to rhizosphere functional plasticity than to taxonomic restructuring. These findings highlight the role of the keystone taxon *Sphingomonas* in maintaining rhizosphere homeostasis, offering a theoretical framework for targeted microbiome engineering to mitigate transplant shock and enhance crop resilience.

## 1. Introduction

Sweet cherry (*Prunus avium* L.) is a highly valued horticultural crop cultivated globally for its nutritional benefits and substantial economic significance [1,2]. In China, the elite cultivar “Hongdeng” is extensively planted due to its superior fruit quality and early maturation. However, *P. avium* is highly sensitive to edaphic and climatic variations. The regional relocation of high-quality cultivars—such as transplanting from the humid coastal climate of Dalian (DL) to the semi-arid inland environment of Hohhot (HS)—often subjects the trees to severe abiotic stresses. This drastic environmental shift typically induces “transplantation shock,” leading to compromised physiological homeostasis, reduced survival rates, and stunted vegetative growth [3,4,5].

While conventional agricultural practices rely heavily on intensive irrigation and fertilization to mitigate these stress responses, the critical role of the rhizosphere microbiome in facilitating plant environmental acclimation has been increasingly recognized [6,7]. Acting as the plant’s “second genome,” the root-associated microbiome actively participates in nutrient acquisition and stress tolerance. The assembly of these complex microbial communities is governed by a dynamic balance of deterministic processes and stochastic processes [8,9].

The intense selective pressures imposed by a transition from a humid to a semi-arid habitat are likely to alter these assembly rules. Comparing the introduced “Hongdeng” (HS) with a locally acclimated reference cultivar, “Summit” (HSY), offers a unique ecological model. HSY serves strictly as an ecological reference to benchmark the baseline microbial and functional characteristics of a successfully stabilized rhizosphere in the target semi-arid habitat, rather than as a genetic control, thereby avoiding misinterpretation of genotypic variations. By defining this ecological benchmark, we can investigate whether translocated plants can restructure a stable and functionally beneficial microbiome analogous to that of indigenous plants. Within these complex networks, specific keystone taxa, particularly drought-tolerant groups such as *Sphingomonas*, are hypothesized to play disproportionately significant roles in maintaining community stability and conferring host resilience under resource-poor conditions [10,11,12,13].

While previous research on fruit tree relocation has extensively utilized 16S rRNA amplicon sequencing to characterize taxonomic shifts, this marker gene approach is inherently limited in its ability to resolve the functional landscape of a community [14,15,16,17,18]. In contrast, metagenomic sequencing provides a direct assessment of the metabolic potential and functional repertoire, enabling a more comprehensive understanding of the mechanisms by which the rhizosphere microbiome facilitates host acclimation to environmental stress. Consequently, a transition to shotgun metagenomic sequencing is imperative to decipher how critical metabolic functions—such as DNA repair, reactive oxygen species (ROS) scavenging, and nutrient transport—are reconfigured under drought and transplant stress. Furthermore, many studies remain limited to demonstrating simple correlations between microbial abundance and plant phenotypes, which often fall short of resolving complex interdependencies. Advanced statistical frameworks, such as structural equation modeling (SEM), offer a robust approach to partitioning the direct and indirect associations among edaphic factors, microbial features, and host physiology, thereby providing insights into the hierarchical structure of these interactions [19,20].

This study investigated the rhizosphere microbiomes of three groups, including the source population in DL, the introduced population in HS, and the local reference (HSY). The research aimed to achieve three primary goals. Firstly, we quantified how regional environmental shifts alter the balance between deterministic and stochastic assembly processes. Secondly, we identified keystone microbial taxa and their associated functional potential in response to semi-arid stress. Thirdly, we constructed a structural equation model to establish the hierarchical links between environmental pressure, the genus *Sphingomonas*, stress-repair pathways, and plant physiological adaptation.

## 2. Results

### 2.1. Plant Physiological Responses to Regional Environmental Shifts

**Figure 1:**
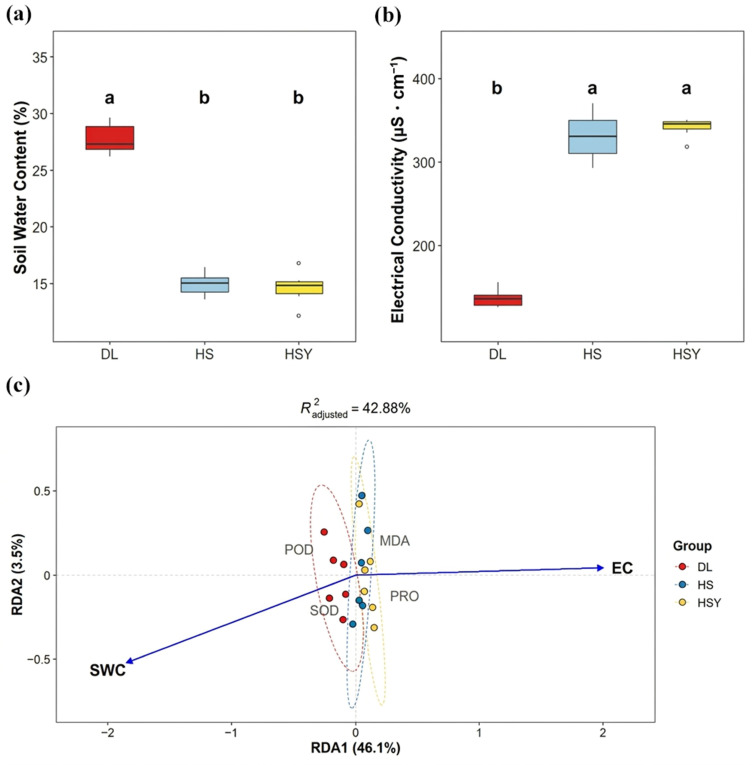
Soil environmental shifts and associated plant physiological responses across distinct climatic regions. (**a**) SWC and (**b**) EC box plots representing core soil physicochemical properties. The significant decrease in SWC and increase in EC from DL to HS and HSY reflect the environmental transition from a humid to a semi-arid habitat. Different lowercase letters above the bars indicate significant differences (*p* < 0.05) based on one-way ANOVA followed by Duncan’s multiple range test (n = 6). (**c**) RDA biplot illustrating the relationships between soil factors (blue arrows) and plant physiological stress indicators (MDA, PRO, SOD, POD). The model explains an adjusted variance ($R_{adjusted}^{2}$) of 42.88%. The overlapping confidence ellipses and clustering of HS and HSY samples demonstrate a physiological convergence between the introduced cherry and the local reference in response to the semi-arid environment.

Moving “Hongdeng” sweet cherry from DL to HS resulted in a significant shift in both soil environmental conditions and the plant’s physiological state. Soil water content (SWC) was significantly lower in the semi-arid HS and HSY groups compared to the humid DL group (Figure 1a; *p* < 0.05). Conversely, soil electrical conductivity (EC) was markedly higher in the Hohhot regions (HS and HSY), confirming a transition to a more saline and water-stressed environment (Figure 1b; *p* < 0.05). These pedological alterations established a distinct selective pressure for both the host plant and its associated microbiota.

The redundancy analysis (RDA) results further demonstrated that these soil properties strongly influenced the physiological adaptations of the sweet cherry trees, with the first two axes explaining 49.6% (RDA1: 46.1%, RDA2: 3.5%) of the total variance (Figure 1c). Compared to the DL group, the HS and HSY samples cluster closely together in the RDA plot. This overlap reveals a physiological convergence between the two groups. The introduced sweet cherry (HS) has adapted to the new environment, making its physiological state comparable to the local reference (HSY). The introduced sweet cherry (HS) and the local reference (HSY) showed distinct physiological responses to this environmental stress compared to the DL group. Specifically, the indicators of oxidative stress and osmotic adjustment—including malondialdehyde (MDA), proline (PRO), superoxide dismutase (SOD), and peroxidase (POD)—were positively correlated with EC and negatively correlated with SWC. This shift illustrates that the trees actively restructured their physiological mechanisms to facilitate survival in the drier, high-conductivity conditions of the introduced site, setting the stage for a corresponding reorganization of the rhizosphere microbiome.

### 2.2. Significant Shifts in Rhizosphere Microbial Diversity and Structural Resilience

**Figure 2:**
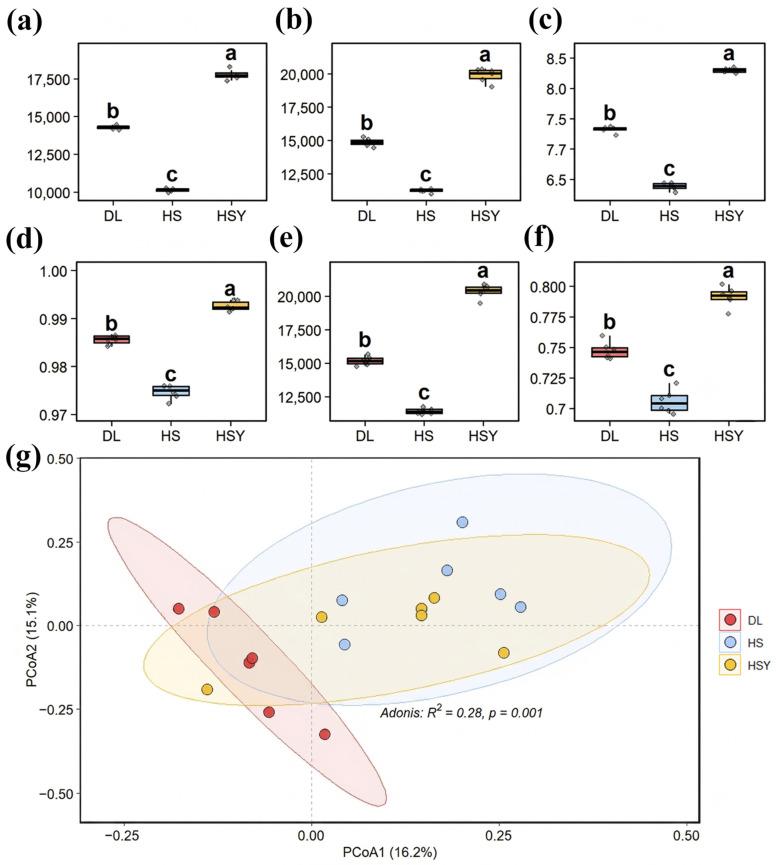
Taxonomic diversity and structure of the rhizosphere microbiome. (**a**) Observed species, (**b**) Chao1, (**c**) Shannon, (**d**) Simpson, (**e**) ACE, and (**f**) Pielou’s evenness alpha diversity metrics of the root microbial communities across DL, HS, and HSY groups. Box plots show the median, quartiles, and outliers. Data were tested using the Kruskal–Wallis test; distinct lowercase letters (a, b, and c) indicate significant differences among groups (*p* < 0.05). (**g**) Beta diversity shown by Principal Coordinate Analysis (PCoA) based on Bray–Curtis distances. The Adonis test (*R*^2^ = 0.28, *p* = 0.001) indicates that the community structure differed significantly among the three groups despite the overlapping ellipses (95% confidence).

We employed metagenomic sequencing to evaluate how the regional environmental transition affected the microbial diversity of the “Hongdeng” sweet cherry rhizosphere. An analysis of alpha diversity indices—including Observed Species, Chao1, Shannon, and Simpson—revealed significant differences among the DL, HS, and HSY groups (Figure 2a–f; *p* < 0.05). Specifically, the indigenous group in the semi-arid region (HSY) exhibited the highest microbial richness and diversity, whereas the introduced group (HS) showed a marked decline compared to the original humid region group (DL). These results, as indicated by the distinct letter markings (a, b, and c), suggest a clear divergence in microbial composition and structural complexity within the root zone following translocation to the new climate.

To assess the divergence in overall community composition, Principal Coordinate Analysis (PCoA) based on Bray–Curtis distances was performed (Figure 2g). The Adonis test results confirmed that the environmental transition significantly reshaped the microbial community structure (*R*^2^ = 0.28, *p* = 0.001). The two primary axes collectively explained 31.3% of the total variance (PC1: 16.2%, PC2: 15.1%). While this proportion is relatively low, it is consistent with the high taxonomic complexity and inherent stochasticity of rhizosphere metagenomes. To verify the robustness of this structural divergence, a sensitivity analysis was conducted using the Aitchison distance matrix, which explicitly accounts for the compositional nature of sequencing data via centered log-ratio (CLR) transformation. This supplementary analysis confirmed a highly significant community separation with remarkably robust explanatory power (Adonis: *R*^2^ = 0.83, *p* = 0.005). The partial overlap of the 95% confidence ellipses in the PCoA, coupled with these robust distance metrics, suggests that while environmental filtering significantly altered the relative abundance of peripheral taxa, a stable core taxonomic framework persisted across different geographic locations. This indicates that the rhizosphere microbiome undergoes targeted structural shifts while maintaining a resilient foundational assembly.

### 2.3. Shifts in Microbial Community Assembly

**Figure 3:**
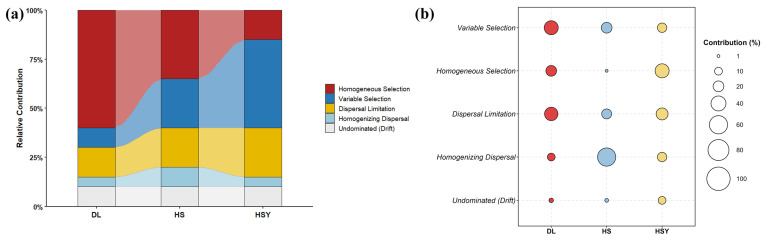
Community assembly processes of rhizosphere microbial communities across different regions. (**a**) Relative contribution of deterministic and stochastic processes. Assembly mechanisms include Variable Selection, Homogeneous Selection (deterministic), Dispersal Limitation, Homogenizing Dispersal, and Undominated/Drift (stochastic). (**b**) Comparative importance of each assembly process across the three sampling sites (DL, HS, and HSY). The size of the bubbles represents the percentage contribution of each process to the community composition.

To understand the mechanisms governing the microbial community structure, we evaluated the relative importance of deterministic and stochastic processes using a null model framework. The assembly of the rhizosphere microbiome was characterized by an interplay of selection, dispersal, and drift, with their relative contributions varying across the three groups (Figure 3).

In the DL group, deterministic processes played a dominant role, primarily through Homogeneous Selection (approximately 60%). This indicates that stable environmental conditions in this group led to a high degree of similarity in community composition. In contrast, the contributions of stochastic processes, such as Dispersal Limitation and Drift, remained relatively low.

When transitioning to the HS and HSY groups, the community assembly patterns changed significantly. In the HS group, the influence of Homogeneous Selection decreased, while Homogenizing Dispersal emerged as a major factor (exceeding 40%), suggesting that high rates of microbial movement facilitated community similarity. In the HSY group, the contribution of Variable Selection increased substantially (near 45%). This shift demonstrates that environmental heterogeneity in the HSY habitat imposed different selective pressures, leading to greater divergence in microbial community structure compared to the other groups. Across all groups, Undominated (Drift) and Dispersal Limitation maintained a consistent but moderate presence, representing the inherent stochasticity in the colonization of the root system.

### 2.4. Identification of Key Microbial Groups

**Figure 4:**
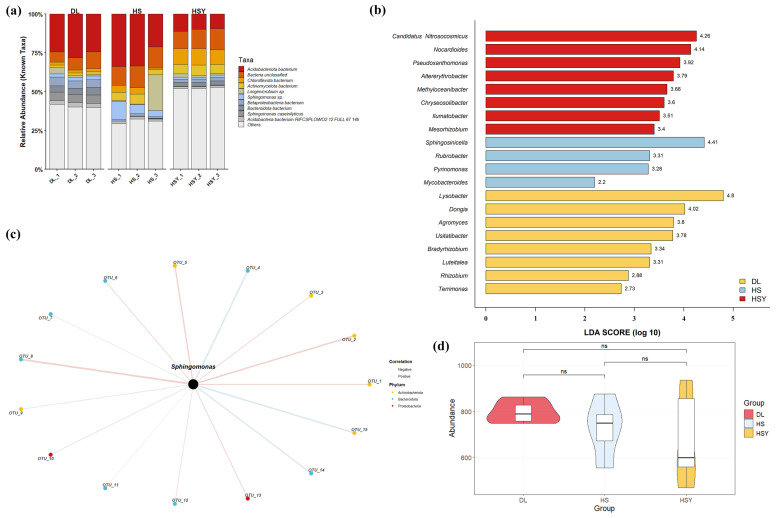
Identification of key microbial groups and their interaction patterns. (**a**) Relative abundance of the top 10 known microbial species across DL, HS, and HSY environments. The stacked bar chart illustrates the overall community composition, with “Others” representing the sum of remaining taxa. (**b**) Identification of microbial taxa showing significant differences across environments via LEfSe analysis. The bar chart displays groups that reached the statistical threshold (LDA score > 3.0, *p* < 0.05), with colors indicating the group where each microbe is most enriched. (**c**) Co-occurrence network highlighting the central role of *Sphingomonas*. Nodes are color-coded by phylum, and lines represent significant relationships between microbes. *Sphingomonas* is identified as a primary hub with numerous connections across the community. To ensure readability, the sub-network highlights the 15 most prominent correlations involving *Sphingomonas*. (**d**) Comparison of the relative abundance of *Sphingomonas* among the three groups. The combined box plot and distribution curve show steady population levels; the “ns” label indicates that differences are not statistically significant (*p* > 0.05), emphasizing that its critical role as a structural network hub is independent of its overall population size.

The initial assessment of the root-associated microbial communities across the DL, HS, and HSY environments shows a consistent foundational structure (Figure 4a). The communities are largely dominated by *Acidobacteriota*, *Chloroflexota*, and various unclassified bacteria.

Although the general composition is similar, the distribution of the top 10 known species exhibits distinct variations in abundance among the three groups, providing a reference framework for identifying the microbes associated with the differences between these environments.

To isolate the microbes playing central roles, a combination of statistical enrichment and network analyses was conducted. The LefSe analysis results identified several microbial groups that were specifically enriched in each of the three environments (Figure 4b). Certain taxa were significantly more abundant in the DL group, while others uniquely characterized the HS and HSY groups. These findings indicate that while the overall community framework remains stable, specific environmental conditions select for distinct microbial signatures.

The co-occurrence network analysis (Figure 4c) results further identified *Sphingomonas* as a keystone node within the rhizosphere microbiome. Based on topological metrics (Table 1), this genus exhibited a high degree of centrality (Average Degree = 45), ranking within the top 5% of all network nodes and functioning as a primary structural hub. Despite this high network centrality, the relative abundance of *Sphingomonas* remained statistically stable across the DL, HS, and HSY groups (Figure 4d, *p* > 0.05). This decoupling of topological importance from numerical dominance indicates that the ecological role of *Sphingomonas* is defined by its connectivity and position within the community architecture rather than its total population size. The LefSe results (Figure 4b) further showed an enrichment of specific stress-tolerant *Sphingosinicella* lineages in the HS group, suggesting that niche specialization—rather than biomass expansion—underpins its contribution to community stability under regional environmental stress.

### 2.5. Enrichment of Stress-Repair Pathways

**Figure 5:**
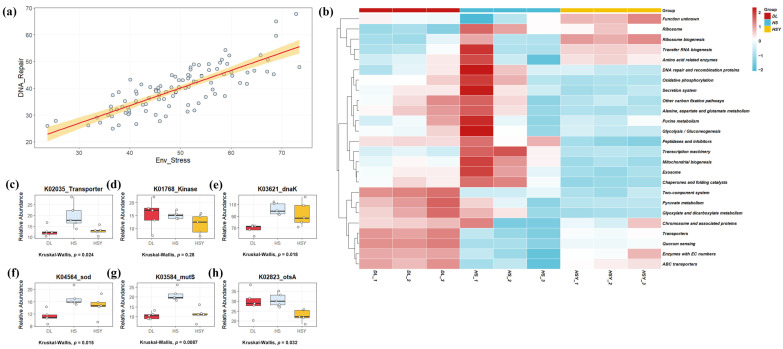
Functional enrichment of stress-repair pathways. (**a**) Linear regression analysis showing a strong positive correlation between E_S and DNA repair pathways. Each data point represents the normalized abundance of a specific KO (KEGG Ortholog) assigned to the DNA repair pathway across individual samples. (**b**) Heatmap of the top 25 functional pathways based on KEGG annotation, highlighting the enrichment of ABC transporters, two-component systems, and repair-related proteins in HS and HSY groups. (**c**) K02035_Transporter, (**d**) K01768_Kinase, (**e**) K03621_*dnaK*, (**f**) K04564_*sod*, (**g**) K03584_*mutS*, and (**h**) K02823_*otsA* showing the relative abundance of key functional genes across the three environments. *p*-values were determined using the Kruskal–Wallis test to evaluate statistical significance.

To evaluate the functional roles of the root-associated microbiome, we analyzed their metabolic pathways using Kyoto Encyclopedia of Genes and Genomes (KEGG) annotations. Although the taxonomic composition remained relatively stable, the microbial functional profiles exhibited significant shifts across different environments. We identified a robust positive correlation between the Environmental Stress index (E_S) and the abundance of DNA repair pathways (*R* = 0.81, *p* < 0.001; Figure 5a).

Further analysis of functional pathways showed that categories related to stress response were significantly altered across environmental groups (Figure 5b). The HS group showed higher abundance of DNA repair and chaperone proteins, whereas ABC transporters and two-component systems were predominantly enriched in the DL group. Statistical tests on key functional genes confirmed this trend (Figure 5c–h). Specifically, the levels of genes tied to stress tolerance and cell repair—such as *dnaK* (chaperone protein), *sod* (superoxide dismutase), and *mutS* (DNA repair)—were significantly higher in the stressed environments.

### 2.6. Integrative Model of Interactions

**Figure 6:**
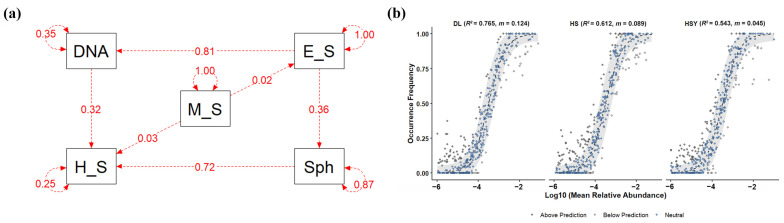
Integrative model of plant–microbiome–environment interactions. (**a**) SEM characterizing the direct and indirect relationships among environmental stress, microbial assembly processes, *Sphingomonas* abundance, stress-repair pathways, and plant physiological adaptation. Path coefficients are indicated next to the arrows; solid lines represent significant paths, while dashed lines indicate non-significant relationships. Model fit indices: $\chi^{2}/df$ = 1.15, *p* = 0.324, CFI = 0.985, RMSEA = 0.038. (**b**) Neutral community model (NCM) for microbial assembly in DL, HS, and HSY groups, showing the fit of the neutral model and the influence of stochastic processes on community structure across different environments.

To synthesize these findings, we integrated environmental data, microbial community assembly patterns, and plant physiological responses using SEM and Variance Partitioning Analysis (VPA) (Figure 6).

The SEM results reveal a structured interaction pathway within the rhizosphere (Figure 6a). Environmental stress significantly modulated the microbial community assembly and enriched specific stress-repair pathways. Notably, the keystone taxon *Sphingomonas* showed a strong connection to these repair functions, which in turn were positively correlated with the host plant’s physiological adaptations, including increased levels of proline (PRO) and superoxide dismutase (SOD). These interactions suggest that *Sphingomonas* and functional pathways act in coordination, collectively enhancing the survival of sweet cherry trees in arid climates.

The neutral community model indicates that environmental shifts altered the balance between stochastic and deterministic processes in microbial assembly (Figure 6b). This shift underscores how external stress reconfigures the rhizosphere microbiome to prioritize functional survival over random colonization.

## 3. Discussion

### 3.1. Physiological Adaptation to the Arid Environment

Relocating fruit trees to a new climate imposes significant environmental stress [21,22,23]. Survival in disparate climatic regions requires rapid physiological adjustments to mitigate environmental stress. Our results showed that the introduced “Hongdeng” population (HS) exhibited significant physiological shifts in response to the high-conductivity and moisture-deficient soils of Hohhot. Specifically, the marked accumulation of PRO and the elevated activities of antioxidant enzymes, such as SOD and POD, suggest a robust activation of osmotic adjustment and ROS scavenging mechanisms [24,25]. These biochemical responses are well-documented strategies for maintaining cellular integrity under water deficit and ionic stress [26,27]. Notably, these physiological modifications led the translocated “Hongdeng” to develop a metabolic profile closely resembling that of the locally adapted “Summit” (HSY). This convergence suggests that the trees underwent systematic metabolic reorganization to stabilize their internal physiological state, rather than simply suffering from transient transplantation shock. Admittedly, these bulk physiological assays only capture a tissue-wide average, which can obscure how specific root cells react to stress. Since recent single-cell studies have revealed significant ‘hidden cellular heterogeneity’ during plant acclimation [28], a logical next step would be to pair this high-resolution host data with our microbiome profiles.

### 3.2. Community Stability and Shifts in Assembly Rules

Extreme environmental shifts frequently disrupt local microbial communities [29,30,31]. However, we observed a resilient pattern within the root microbiome. Although the total richness of microbial species decreased in the introduced trees (HS), the core taxonomic groups remained stable. The underlying reason for this stability lies in a shift in microbial assembly rules. In the humid Dalian soil (DL), the environment favored similar microbial groups through Homogeneous Selection. Conversely, in the introduced HS group, the contribution of Homogenizing Dispersal increased significantly. This enhancement of Homogenizing Dispersal under semi-arid stress may initially seem counterintuitive, but it likely reflects a localized “mass effect” mediated by the host plant. Under acute transplantation shock, sweet cherry roots likely exude a uniform profile of stress-signaling compounds, creating a highly homogenized selective pull across the immediate root zone. This strong, uniform recruitment can largely counteract local edaphic micro-heterogeneity, fostering high rates of microbial exchange and structural similarity within the rhizosphere as specific stress-responsive taxa rapidly colonize the available space. Furthermore, the recent literature on microbiome resilience highlights that such shifts in assembly processes frequently underpin functional redundancy [32,33]. The community maintains its taxonomic foundation and critical metabolic outputs despite the loss of peripheral alpha diversity, utilizing this functional plasticity to sustain ecosystem services under novel environmental constraints.

### 3.3. The Ecological Role of Sphingomonas

Our network analysis results identified *Sphingomonas* as a keystone taxon in the co-occurrence network, yet its overall relative abundance did not show statistically significant changes across the DL, HS, and HSY groups (Figure 4d). This indicates that the ecological importance of this taxon is determined not by its relative abundance, but by its high centrality within the network topology. High network centrality indicates that *Sphingomonas* is vital for maintaining the community structure [34,35]. Consistent with the definition of a keystone taxon, its ecological impact is independent of its relative abundance [36,37]. Therefore, its stable population size across groups does not mean it is less important. Recent studies confirm that such central nodes stabilize the community under stress without increasing in number [38]. In the network analysis, *Sphingomonas* exhibited high connectivity, showing significant co-occurrence relationships with multiple distinct microbial taxa. The LEfSe results also revealed that while the total abundance of the genus remained stable, specific stress-tolerant lineages within *Sphingomonas* significantly increased in abundance under semi-arid environmental pressure.

Physiologically, members of this genus possess characteristics that facilitate their survival in the semi-arid and high-conductivity soil environment of Hohhot. Their cell envelopes are rich in sphingolipids, a structural feature that maintains cell membrane integrity and reduces the leakage of intracellular contents under osmotic and oxidative stress [39,40]. Furthermore, *Sphingomonas* can secrete exopolysaccharides (EPS), which alter the physicochemical properties of the rhizosphere soil [41].

*Sphingomonas* utilizes robust endogenous antioxidant systems to scavenge root-derived reactive oxygen species (ROS), ensuring survival amidst localized oxidative bursts [42]. These combined physiological traits—membrane sphingolipids, EPS secretion, and antioxidant defenses—potentially buffer the immediate rhizosphere. This microenvironmental conditioning may serve to shield stress-sensitive microbial neighbors from severe desiccation and salinity [43]. The high network centrality of *Sphingomonas* reflects its function as a structural anchor. It stabilizes the microbiome and sustains critical nutrient cycling, providing a resilient biological foundation for the host plant’s adaptation to semi-arid stress [44,45,46].

### 3.4. Functional Synergy Between the Microbiome and the Host Plant

Our analysis identified a robust positive correlation (*R* = 0.81, *p* < 0.001) between $E_{S}$ and the abundance of microbial DNA repair pathways. The significant enrichment of core functional genes, specifically *dnaK* (chaperone protein), *sod* (superoxide dismutase), and *mutS* (DNA repair), indicates an enhanced genomic potential within the rhizosphere microbiome to mitigate proteotoxic and oxidative damage under semi-arid conditions [47,48]. In the high-conductivity and moisture-deficient soils of Hohhot, these genes likely facilitate the stabilization of protein folding and the neutralization of superoxide radicals, thereby maintaining microbial metabolic activity despite osmotic stress.

This microbial stress-response capacity extends its influence to the host–soil interface. The localized secretion of EPS and the enzymatic degradation of ROS by taxa such as *Sphingomonas* can modify the immediate rhizospheric environment [49]. These processes potentially facilitate the sequestration of excess ions and buffer the root system against oxidative bursts [50,51]. The upregulation of ROS-scavenging enzymes allows the rhizosphere microbiome to regulate the local redox state. Rather than merely acting as a passive sink, this microbial regulation aligns with modern redox-signaling frameworks, where ROS serve as both damaging agents and essential adaptive signals [52]. By fine-tuning this redox balance, the microbiome protects root tissues and maintains the plant’s capacity for water and nutrient uptake [53]. In parallel, enhanced DNA repair pathways enable these microbes to persist under severe soil stress [54]. The sustained presence of these communities creates a protective interface around the roots, which supports the long-term establishment of the host plant [55]. Rather than a simple synergistic adaptation, this evidence suggests a potential microbially mediated mechanism for alleviating oxidative stress that stabilizes the physicochemical niche of the “Hongdeng” sweet cherry. These findings provide a theoretical basis for utilizing native keystone taxa in microbiome-based interventions [10,56]. Strategic management—such as the pre-inoculation of drought-tolerant *Sphingomonas* strains or the application of specific carbon sources to stimulate indigenous repair pathways—offers a promising avenue for mitigating transplantation shock and improving orchard establishment in arid or salinized regions [57,58].

**Figure 7:**
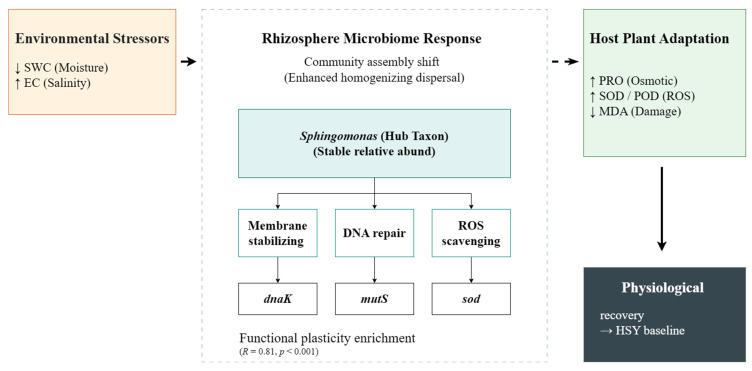
Conceptual model of the microbiome-associated adaptation of *Prunus avium* to semi-arid stress. Edaphic stressors (decreased soil water content, SWC; increased electrical conductivity, EC) shift the assembly processes of the rhizosphere microbial community. Within this altered network, the keystone genus *Sphingomonas* maintains structural stability and enriches specific functional pathways in the root zone, including cellular membrane stabilization (*dnaK*), DNA repair (*mutS*), and reactive oxygen species (ROS) scavenging (*sod*). This microbial functional response is associated with improved host stress tolerance, indicated by reduced lipid peroxidation (MDA) alongside enhanced osmotic (PRO) and antioxidant (SOD, POD) defenses. Ultimately, these coordinated interactions support the physiological acclimation of the host plant toward the local reference baseline. The dashed arrow denotes a proposed ecological link rather than a validated causal relationship.

To summarize these interactions, we proposed a conceptual model illustrating how the rhizosphere microbiome and the keystone taxon *Sphingomonas* support sweet cherry adaptation to semi-arid stress (Figure 7). Under environmental pressures of low soil moisture (SWC) and high salinity (EC), deterministic selection shapes the rhizosphere community structure. As a prominent keystone taxon, *Sphingomonas* stabilizes the co-occurrence network and enriches specific stress-response pathways, including cellular membrane stabilization (*dnaK*), DNA repair (*mutS*), and ROS scavenging (*sod*). These microbial functions are associated with improved host tolerance, as indicated by decreased leaf MDA levels alongside increased PRO content and enhanced SOD and POD activities. Consequently, these coordinated responses support the physiological acclimation of translocated *P. avium* toward the local reference baseline (HSY).

### 3.5. Limitations and Future Directions

The ecological relationships inferred by our structural equation modeling (SEM) remain fundamentally correlative due to the observational nature of the field data. A primary limitation is the lack of in vivo inoculation or functional verification for the identified keystone taxon. Although network analysis highlighted *Sphingomonas* as a structural anchor, whether this genus actively drives plant acclimation or simply tolerates the environmental pressures remains to be empirically validated. Future studies should employ targeted manipulation experiments, such as single-strain inoculations or synthetic microbial communities (SynComs) using knockout strains deficient in key stress-response genes, to establish direct causal links.

These modeled interactions also likely involve bidirectional feedback loops that could not be fully decoupled within the scope of this study. While microbial recruitment assists the host, plant-mediated responses under semi-arid stress—such as altered root exudation—simultaneously restructure the rhizosphere community. Because this work lacks empirical data on root exudates and soil metabolites, the specific metabolic mechanisms underlying these host-microbe interactions could not be resolved. Integrating root exudate profiling with host transcriptomics and metabolomics represents a necessary step to characterize these underlying signaling pathways.

The current sampling strategy represents a cross-sectional snapshot restricted to the peak vegetative stage, leaving long-term temporal successional dynamics uncharacterized. Consequently, the rate of functional shifts and their stability across contrasting seasons or successive years remain unknown. Evaluating the consistency and sustainability of microbiome-mediated adaptation in translocated perennial crops will ultimately require multi-year longitudinal sampling paired with these multi-omics approaches.

## 4. Materials and Methods

### 4.1. Experimental Site and Plant Materials

We focused on the ecological adaptation of *P. avium* during its regional introduction across contrasting climatic zones. Specifically, we transplanted cherry trees from Dalian (38°54′ N, 121°38′ E)—a coastal area with a humid climate—to Hohhot (40°48′ N, 111°41′ E), an inland region with a semi-arid climate and notable temperature fluctuations.

Three experimental groups were established for comparative analysis: the source *P. avium* population in Dalian (cultivar “Hongdeng”), the “Hongdeng” population introduced to Hohhot, and the local reference cultivar in Hohhot (HSY, cultivar “Summit”). All test trees were six years old and grafted onto Gisela 6 rootstocks, ensuring a uniform rootstock background to isolate the scion’s response to the contrasting environments.

To avoid interference from external variables, the HS and HSY groups in Hohhot were planted in the same experimental orchard. We applied synchronized field management measures, including irrigation, fertilization, and pest control, to ensure uniform growing conditions. This experimental design ensured that any observed differences in rhizosphere properties and microbial communities were primarily attributed to cultivar-specific traits and long-term environmental adaptation, rather than variations in field management practices [59].

The locally acclimated cultivar “Summit” (HSY) was included in the experimental design not as a genetic control for the introduced “Hongdeng” (HS), but as an ecological reference for the regional semi-arid environment. By comparing HS with HSY, this study aimed to assess whether the translocated cultivar could assemble a rhizosphere microbiome that is structurally and functionally analogous to that of a locally established population under identical pedoclimatic conditions.

The “Hongdeng” trees were translocated from Dalian to Hohhot and allowed to acclimate for two consecutive growing seasons prior to sampling. This timeframe ensured that we captured mid-to-long-term environmental acclimation rather than the acute mechanical damage of transplantation. While this cross-sectional design captures a stabilized, multi-year acclimation state rather than the immediate velocity of post-transplantation microbial succession, it establishes a reliable baseline for mid-term adaptation. Furthermore, the locally cultivated “Summit” (HSY) was exclusively utilized as a regional ecological benchmark representing the climax rhizosphere equilibrium in Hohhot’s semi-arid soil. We explicitly refrain from direct genetic comparisons between HS and HSY; rather, HSY serves to illustrate the baseline microbial landscape shaped by the local pedoclimatic conditions.

### 4.2. Sample Collection and Physiological Index Determination

Rhizosphere soil samples were collected during the peak vegetative growth period. For each group (DL, HS, and HSY), six trees were randomly selected. After vigorously shaking off bulk soil, the soil tightly adhering to the root surface (1–2 mm in thickness) was collected as rhizosphere soil using sterile brushes. Samples were immediately flash-frozen in liquid nitrogen and stored at −80 °C [60].

To assess the soil microenvironment, core physicochemical properties including SWC and EC were measured. SWC was determined using the gravimetric method after drying the soil at 105 °C to a constant weight, and EC was measured in a 1:5 soil-to-water extract using a conductivity meter (DDS-307; INESA Scientific Instrument Co., Ltd., Shanghai, China) [61]. For plant physiological responses, leaves were sampled to determine the levels of MDA and PRO and the activities of SOD and POD using standard commercial assay kits (Solarbio Science & Technology Co., Ltd., Beijing, China) following the manufacturer’s instructions [62,63].

### 4.3. Metagenomic Sequencing and Bioinformatic Analysis

Total microbial genomic DNA was extracted from 0.5 g of each rhizosphere soil sample using the E.Z.N.A.^®^ Soil DNA Kit (Omega Bio-tek, Norcross, GA, USA) according to the manufacturer’s protocols. To ensure the fidelity of DNA-based metagenomic profiling, RNA was rigorously removed during the nucleic acid extraction phase using RNase A treatment. Shotgun metagenomic sequencing (150 bp paired-end) was performed on the Illumina NovaSeq 6000 platform (Illumina, Inc., San Diego, CA, USA) by LC-Bio Technology Co., Ltd. (Hangzhou, China), generating an average sequencing depth of 10.0 Gb of clean data per sample.

Raw reads were filtered using fastp software (version 0.23.2) to obtain high-quality clean data, and clean reads were aligned to the *P. avium* reference genome using Bowtie2 (version 2.4.4) to eliminate host DNA [64,65]. High-quality sequences were assembled using MEGAHIT (version 1.2.9) [66]. This de novo assembly yielded an average of 335,287 contigs (≥500 bp) per sample, with a mean N50 length of 1062 bp, ensuring sufficient assembly contiguity for accurate downstream functional annotation. Taxonomic classification was conducted using DIAMOND software (version 2.0.15) by aligning protein sequences against the NR_meta database [67]. To identify key microbial groups driving environmental differences, LEfSe (version 1.1.2) was performed (LDA score > 3.0, *p* < 0.05) [68]. To characterize microbial interactions, a co-occurrence network was constructed using Spearman’s rank correlation coefficients, retaining only robust associations with |*ρ*| > 0.6 and *p* < 0.05 [69]. Topological properties were calculated via the Gephi platform (version 0.9.2), and keystone taxa were objectively identified as nodes ranking within the top 5% of both degree and betweenness centrality [70]. For functional profiling, metagenomic sequences were aligned against the KEGG database using DIAMOND (version 0.9.24) [71]. Functional orthologs (KOs) were assigned based on high-scoring hits (E-value < 1 × 10^−5^), with subsequent analysis focusing on metabolic pathways involved in DNA repair, antioxidant defense, and osmotic regulation.

### 4.4. Microbial Community Assembly and Integrative Statistical Analysis

To quantify the assembly mechanisms of the rhizosphere microbiome, the β-nearest taxon index (βNTI) and Raup–Crick metric (${RC}_{bray}$) were calculated using the “picante” package (version 1.8.2) [72,73]. These indices were utilized to partition the relative influence of deterministic and stochastic processes. To quantify the relative influence of stochastic processes on microbial assembly, the Sloan NCM was implemented [74]. Calculations were performed in R (version 4.4.3) using the “minpack.lm” (version 1.2-4) and “Hmisc” (version 5.1-1) packages, with the model’s fit evaluated based on the coefficient of determination (*R*^2^) [75,76]. The migration rate (*m*) was further estimated to assess Dispersal Limitations across the DL, HS, and HSY groups. This approach allowed for a rigorous assessment of how the environmental transition altered the balance between deterministic selection and stochastic drift in the rhizosphere.

To assess the cumulative impact of edaphic stressors, $E_{S}$ was constructed [77]. SWC and EC were selected as the core components because they capture the primary osmotic and desiccation pressures driving transplantation stress in the semi-arid region. Other edaphic factors, such as soil pH and nutrient content, were excluded because uniform orchard management maintained them at relatively homogeneous, non-stressful levels across plots, precluding them from acting as selective environmental filters. Meanwhile, temperature was excluded as it reflects macroclimatic variation rather than local root-zone edaphic stress. Collinearity between SWC and EC was negligible, as indicated by a Variance Inflation Factor (VIF) of 1.63, which is well below the conservative threshold of 5.0, confirming their independence. Specifically, SWC values were inverted to reflect increasing stress with decreasing moisture, and both inverted SWC and EC were normalized using Z-score standardization. The $E_{S}$ for each sample was defined as the arithmetic mean of these standardized scores.

Alpha diversity metrics, including Observed species, Chao1, Shannon, Simpson, ACE, and Pielou’s evenness, were calculated using the “vegan” package (version 2.6-4) in R to assess within-sample taxonomic complexity, with statistical differences among groups evaluated by the Kruskal–Wallis test [78]. Differences in microbial community structure were evaluated via PCoA based on Bray–Curtis distances, with statistical significance determined by the Adonis test (PERMANOVA). To validate the robustness of these beta diversity findings, a sensitivity analysis was performed using the Aitchison distance matrix, following a CLR transformation of the taxonomic abundance data using the vegan package. The structural interdependencies among environmental variables, microbial features, and host physiology were analyzed using SEM and VPA [79]. VPA was performed using the “vegan” package to quantify the explanatory power of distinct variable sets. The SEM was constructed using the “lavaan” package (version 0.6-16) to evaluate hypothesized pathways linking $E_{S}$, community assembly processes, keystone taxa (*Sphingomonas*), stress-response pathways, and plant physiological indicators. The genus *Sphingomonas* was incorporated as the core biotic variable based on both network topology and ecological relevance. In our analysis, it emerged as a prominent keystone taxon, ranking within the top 5% for both degree and betweenness centrality. Furthermore, this group is functionally characterized by robust desiccation-tolerance mechanisms and the capacity to mitigate rhizosphere osmotic stress via plant-growth-promoting traits [35,80]. Model fitness was assessed based on the Chi-square test ($\chi^{2}$), Comparative Fit Index (CFI), and Root Mean Square Error of Approximation (RMSEA).

## 5. Conclusions

The transplantation of “Hongdeng” sweet cherry to a semi-arid environment imposes substantial edaphic stress, profoundly shaping the rhizosphere microbiome. Soil moisture and salinity act as primary environmental filters, shifting community assembly from stochastic drift toward deterministic selection. This transition is characterized by a reduction in microbial network complexity while enriching specific keystone taxa, notably *Sphingomonas*, which are associated with enhanced desiccation tolerance and osmotic regulation. Concurrently, the upregulation of functional pathways related to stress repair—including ROS scavenging and DNA repair—maintains microbial viability, potentially contributing to a biological buffer against environmental toxicity in the root zone.

However, the ecological relationships identified in this observational study are correlative. While structural equation modeling suggests a link between microbial shifts and host adaptation, definitive causal inference requires further experimental validation. Future research should employ synthetic microbial communities (SynComs) and in vivo inoculation trials using knockout strains to verify the underlying functional genes. Furthermore, integrating multi-year dynamic monitoring will help to elucidate the temporal successional dynamics of these interactions. Characterizing these specific pathways will ultimately support the development of targeted microbial inoculants to improve the resilience of translocated perennial crops.

## Figures and Tables

**Table 1:** Taxonomic classification, mean relative abundance, and network topological metrics of key microbial taxa identified in the co-occurrence network.

Genus	Phylum	Mean Relative Abundance (%)	Degree Centrality
*Sphingomonas*	*Proteobacteria*	11.70%	45
*Acidobacteriota*_unclassified	*Acidobacteriota*	21.80%	41
*Altererythrobacter*	*Proteobacteria*	0.48%	41
*Algoriphagus*	*Bacteroidota*	0.12%	41

## Data Availability

The data presented in this study are available on request from the corresponding author. The data are not publicly available due to ongoing subsequent research and intellectual property protection within the project group.
